# Shrinking Your Deictic System: How Far Can You Go?

**DOI:** 10.3389/fpsyg.2020.575497

**Published:** 2020-12-02

**Authors:** Mila Vulchanova, Pedro Guijarro-Fuentes, Jacqueline Collier, Valentin Vulchanov

**Affiliations:** ^1^Language Acquisition and Language Processing Lab, Department of Language and Literature, Norwegian University of Science & Technology, Trondheim, Norway; ^2^Departamento de Filología Española, Moderna y Clásica, Universidad de Islas Baleares, Palma, Spain; ^3^School of Psychology, University of East Anglia, Norwich, United Kingdom

**Keywords:** demonstratives, bilingualism, language attrition, norwegian, spatial memory game, spanish sample

## Abstract

Languages around the world differ in terms of the number of adnominal and pronominal demonstratives they require, as well as the factors that impact on their felicitous use. Given this cross-linguistic variation in deictic demonstrative terms, and the features that determine their felicitous use, an open question is how this is accommodated within bilingual cognition and language. In particular, we were interested in the extent to which bilingual language exposure and practice might alter the way in which a bilingual is using deictic demonstratives in their first language. Recent research on language attrition suggests that L2 learning selectively affects aspects of the native language, with some domains of language competence being more vulnerable than others. If demonstratives are basic, and acquired relatively early, they should be less susceptible to change and attrition. This was the hypothesis we went on to test in the current study. We tested two groups of native Spanish speakers, a control group living in Spain and an experimental group living in Norway using the (Spatial) Memory game paradigm. Contra to our expectations, the results indicate a significant difference between the two groups in use of deictic terms, indicative of a change in the preferred number of terms used. This suggests that deictic referential systems may change over time under pressure from bilingual language exposure.

## Introduction

Demonstratives are function words typically used to refer to physical, concrete entities in a real-world speech situation. Utterance of the demonstrative, often accompanied by a pointing gesture (Bühler, 1934; Diessel, 1999, 2006; Levinson, 2004), has an important communicative upshot. It aims to focus the attention of the addressee on a particular entity in the shared perceptual or visual field of the interlocutors. Languages around the world differ in terms of the number of adnominal and pronominal demonstratives they require (Diessel, 1999), as well as the factors that impact on their felicitous use. Distance from the deictic center (i.e., the speaker) has been identified as the most common feature encoded in demonstratives cross-linguistically (Lyons, 1977; Anderson and Keenan, 1985; Diessel, 1999). Following recent in-depth empirical and experimental research, this tradition has been called into question, often referred to as the “spatial bias” in accounts of demonstratives (Levinson, 2018). Thus, in addition to distance from speaker, and/or hearer, visibility, ownership, possibility to interact with the reference object and other features of the speaker-hearer constellation have been shown to be relevant for deictic term use, even in a language like English, which does not encode these features lexically (Coventry et al., 2008, 2014; Levinson et al., 2018).

Given the cross-linguistic variation in deictic demonstrative terms, and the features that determine their felicitous use, an open question is how this is accommodated within bilingual cognition and language. In particular, we are interested in the extent to which daily immersive exposure to a second language and practice might alter the way in which bilingual speakers are using deictic demonstratives in their first language. Recent research on language attrition suggests that L2 learning selectively affects aspects of the native language, with some domains of language competence being more vulnerable than others (Jakobson, 1941; de Bot and Weltens, 1991; Keijzer, 2007). Native language vulnerability is subject to individual variation, and specific factors in the bilingual speaker’s background, such as level of education, literacy etc. (Köpke, 2007). At the same time, it has been shown that the more robust aspects of language are those that are typically acquired early and sub-serve basic language functions (Jakobson, 1941; Keijzer, 2007). If demonstratives are basic (Dixon, 2003), and acquired relatively early (Clark, 1978; Diessel, 2006), they should be less susceptible to change and attrition. This was the hypothesis we went on to test in the current study.

The languages in the current study are a three-term language, Spanish, and a two- term language, Norwegian. Diessel (1999) and Dixon (2003) provide a comprehensive survey of cross-linguistic variation in relation to the system of demonstratives and the parameters affecting the choice of demonstratives in specific contexts. In Diessel’s schematization (2005, 2013), for instance, a two-termed proximal/distance contrast system has a higher frequency (54.4%) than the three-termed contrast (37.4%), and other combinations of demonstratives (8%). In addition, within the frame of proximal/distal opposition, the distance-oriented system is the most widespread (two thirds of the languages analyzed; Diessel, 2005, 2013) in comparison to the person-oriented system.

Spanish features a tripartite demonstrative system with three elements (*este*, *ese*, and *aquel*) (Jungbluth and Da Milano, 2015), which can inflect for gender and number and are used adnominally. In addition, Spanish has three demonstrative pronouns (*esto*, *eso*, and *aquello*), which do not inflect and have, nevertheless, been traditionally labeled as neuter demonstrative pronouns in the Spanish grammatical tradition (although there is not clearly a neuter grammatical gender in Spanish *per se*). The Spanish demonstrative terms are commonly characterized as conveying different degrees of distance with respect to the deictic center (the speaker): *este* (“this”) is proximal, *ese* (“that”) medial, and *aquel* (“that yonder”) is the distal demonstrative of the tripartite system. The Spanish demonstrative system, can thus be seen as gravitating toward an egocentric, distance-oriented preference usage, which accounts for the proximal, medial and distal forms in relation to the speaker, with little or no consideration of the position of the hearer (Diessel, 1999; Jungbluth, 2003; Coventry et al., 2008; Jungbluth and Da Milano, 2015). This is also consistent with Hottenroth (1982) who suggests that the “proximal-medial-distal form designates increasingly remote concentric circles around the speaker” (p. 133). Jungbluth (2003); Coventry et al. (2008), and Jungbluth and Da Milano (2015) presented a more detailed description of the Spanish demonstrative system, taking into account the effect of the hearer’s position in the choice of demonstratives. Jungbluth (2003) and Jungbluth and Da Milano (2015), for instance, suggested a dual-oriented system of interaction with three possible conditions (“constellations”) with respect to the hearer: face-to-face, side-by-side, and face-to-back. During semi-naturalistic performances, Spanish monolingual speakers preferred a distance-oriented system in a side-by-side condition, a person-oriented system in a face-to-face condition and both a person-oriented and a distance-oriented system in a face-to-back condition. Coventry et al. (2008) provide experimental evidence that hearer position impacts on the use of the three terms, and interacts with distance.

Norwegian is a two-term system. Traditionally, the demonstrative pronouns *denne* and *den* have been considered to reflect the contrast between proximal (*denne*) and distal (*den*) object locations (Faarlund et al., 1997). However, the modern colloquial language uses a spatial adverb [*her* (*here*) and *der* (*there*)] as a reinforcement of both *denne* (proximal) and *den* (distal), thus yielding the so-called complex demonstrative forms *den/denne her* (*this here*) (Johannessen, 2006). This possibility comes to suggest that the form *den*, originally assumed to be distal, has evolved into a neutral form rather than signaling distance (Halmøy, 2016). This is further confirmed by the possibility of combining *den* with the distal adverb *der* (*there*), with *den der* meaning “that one over there.” Adverbs denoting location have been the source of reinforcing expressions in several languages world-wide. Furthermore, when a demonstrative adverb is used adnominally, it usually does not function as a modifier of the noun, but rather as a reinforcement of the co-occurring demonstrative determiner. Vindenes (2018) argues that speaker strategies that are used to achieve joint attention are particularly important mechanisms in the (diachronic) process of reinforcement of demonstratives, also evidenced in the Modern Norwegian situation. While Spanish has been studied experimentally, to our knowledge there is no such research on Norwegian.

Dixon (2003) points out that a three-term system of demonstratives might convey either a relative distance (i.e., near, mid and far) or relate to the participant (i.e., near the speaker, near the hearer, near neither), but also to height, stance, visibility as well as elevation and movement (Diessel, 1999; Breunesse, 2020). Other parameters affecting the choice of demonstratives may refer to perspective-taking (e.g., for Turkish, Küntay and Özyürek, 2005), sociocentric proximity (Stevens and Zhang, 2013, 2014; Peeters et al., 2015), semantic features (Rocca et al., 2019), ownership, visibility, and familiarity of referent (Coventry et al., 2014), and proximity/distance of referent in relation to both speaker and hearer (i.e., Spanish, Catalan, and Japanese, Diessel, 1999; Jungbluth, 2003; Coventry et al., 2008).

Given these considerations, the difference between the Spanish and Norwegian adnominal/pronominal demonstrative systems mainly lies in the morpho-lexical choice of demonstrative term, and the number of such terms, while both systems might equally well reflect other semantic distinctions, as documented in extant research.

In the current study we were interested in the extent to which a subsequently acquired two-term system (Norwegian) might impact on the original three-term L1 system (Spanish) in adult language users. Our predictions were that closed-class systems of the deictic type are not easily attrited. However, we did expect subtle deviations from the native Spanish system in terms of specific distinctions (e.g., distance magnitude), and we expected this effect to be attributable to length of stay in Norway. In line with Coventry et al. (2008) we also expected position of hearer to influence participants’ responses.

## Materials and Methods

### Participants

Participants in the experimental group (*Spanish Living in Norway*, henceforth (SLiN)) were 20 adult native speakers of Spanish who had lived in Norway for work or study on average 110,4 months. 2/3rds of the SLiN participants had attended language courses or had experience from Norwegian education, while 1/3rd indicated that they had learned Norwegian naturalistically. Twelve participants rated their level of proficiency in Norwegian as advanced-to-near native, and only two assessed their level as beginners, which reflects advanced knowledge of Norwegian. In addition, all participants (with one exception) stated that they used both languages equally on a daily basis, with some prevalence for Norwegian. They were recruited via various channels, social media, university networks and via social contact. All participants provided signed informed consent prior to the study. Approval for the study and for collecting and storing the data was obtained from the Norwegian Da ta Protection Service (NSD). All SLiN participants had had their first exposure to Norwegian [Age of Arrival (AoA)] after age 20 years. For this reason, we used length of stay as predictor in the analyses.

The control group [*Spanish Living in Spain*; henceforth (SLiS)] comprised *N* = 30 (*MA* = 23.5; *SD* = 5.88; female = 18) native speakers of Castilian Spanish recruited at Universidad de Islas Baleares. Approval for the study and for collecting and storing the data was obtained from Comité de Ética de la Investigación (Universidad de Islas Baleares), and the School of Psychology Ethics Committee at the University of East Anglia as part of a bigger cross-linguistic study. All participants were matched for socioeconomic and educational background. The speakers who volunteered to take part in this study and, therefore, did not get any economic compensation for participation, were residents in Spain at the time of testing.

### Stimuli

**FIGURE 1:**
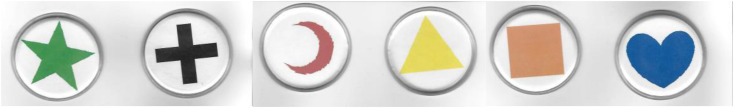
Images of the disks. From left to right, the disks presented the following images: a green star, a black cross, a red moon, a yellow triangle, an orange square and a blue heart.

**FIGURE 2:**
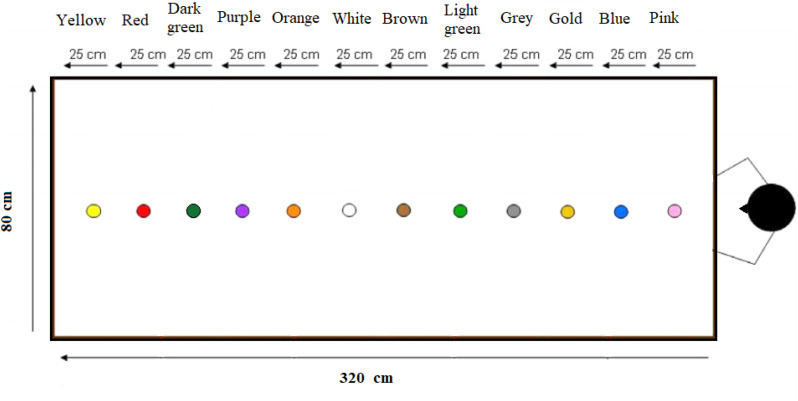
Experiment set up. We used six positions: pink (1st position at 25 cm), blue (2nd position at 50 cm), brown (3rd position at 150 cm), white (4th position at 175 cm), red (5th position at 275 cm) and yellow (6th position at 300 cm). The space could be divided in three subspaces depending on the participants’ arm reach: one peri-personal space, within participants’ arm reach, and two extra-personal subspaces, out of reach.

Participants were tested with the Spanish version of the (Spatial) Memory game (Gudde et al., 2018). The memory game paradigm is a behavioral procedure to explore the relationship between language, spatial memory, and object knowledge and has already been widely used in cross-linguistic research. In two different versions of the paradigm, spatial language use and memory for object location are tested under different, experimentally manipulated conditions. The current study employed only the spatial language use version of the paradigm. Participants were tested in naming markers placed on a table at different distances from the participant (= speaker). In one set up the experimenter (= hearer) was seated next to the participant, and in another, opposite to the participant. We elicited the production of demonstratives by locating six circular plastic disks on top of a conference table. The disks were 6 cm wide and presented different sketched images (see Figure 1). The experimenter located the disks on top of 12 colored dots equally distributed on the table (320 ^∗^ 80 cm, see Figure 2). The table was covered by a black cloth. We used the following 6 locations to locate the disks: 25, 50, 150, 175, 275, and 300 cm.

Participants in the study were instructed to use *este*, *ese*, and *aquel* for the Spanish version of the experiment and *den her* (*this here*) and *den der* (*this there*), for the Norwegian version.

### Procedure and Design

During the experiment, the participants sat at the table (within 3 cm distance), in front of the line marked by the colored dots (40 cm). The experimenter sat either laterally or frontally with respect to the participant. We instructed the participant to memorize the position of the disks that the experiment was locating on top of the dots. To help the memorization process, (s)he had to use a bimodal production: gestural and verbal. Every time the experimenter sat after locating the disk, the participant had to point at the disk (i.e., gestural performance), without standing up or touching the table. In addition, the participant had to produce a sentence consisting of three elements (i.e., verbal performance): a demonstrative, the color and the image in the disk (i.e., this/that red moon). Every time the participant performed the gestural and verbal production, the experimenter stood up to locate the subsequent disk on the list. The trials presented random breaks with memory questions regarding the last position of one or more disks. The total amount of trials was 36 per participant divided in two sub-sessions of eighteen trials each. On eighteen trials the experimenter sat next to the participant [laterally and on the remaining eighteen trials opposite the participant (frontally)]. We counterbalanced the order of presentation of the stimuli, the locations of the discs on the dots, as well as the position of the experimenter to avoid any effect of order.

The whole session, from welcoming to debriefing was conducted in the language of testing by the experimenter.

For the purposes of indirect comparison, we also tested a group of adult Norwegian native speakers living in Norway (*N* = 23; *MA* = 23; *SD* = 2.87; female = 11) which was part of a bigger cross-linguistic study (Coventry, in preparation). Approval for the study was obtained by the University of East Anglia. The participants had similar educational and socio-economic backgrounds.

## Analysis and Results

### Descriptives Before Merging the Data

**FIGURE 3:**
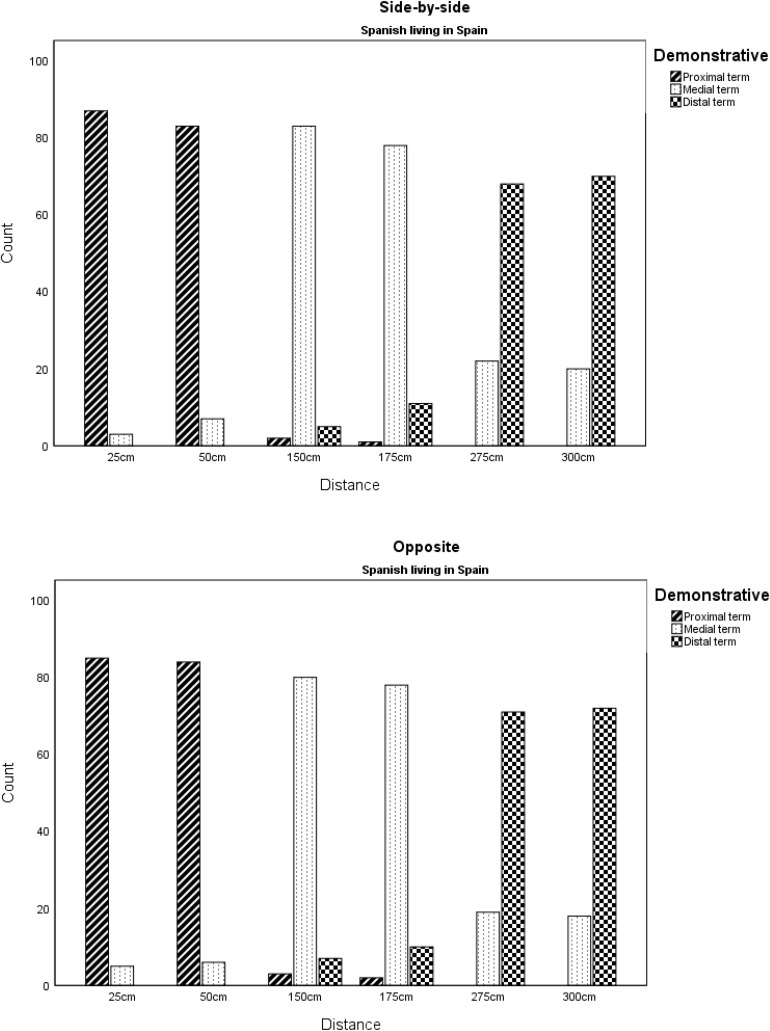
Demonstratives by distance and hearer position for Spanish speakers living in Spain.

The Spanish Living in Spain (SLiS) group used the three terms according to distance from speaker regardless of position of hearer. Thus, the proximal term was used exclusively to name the two closest distances (25 and 50 cm), the distal term was used exclusively to name the two outmost distances (275 and 300 cm), while the medial (third) term was used for the medial positions (150 and 175 cm). This was not the case for the Spanish Living in Norway (SLiN) group, whereby the most prevalent term used was the medial term (*ese*) regardless of distance from speaker/hearer at a total of 420 times (58.3%). Thus, overall, the Spanish Living in Norway used *ese* more than those living in Spain (58.3 vs. 38.3%), with minimal reduction in *este* (27.8 vs. 32.1%) and a notable drop in the use of *aquel/aquella* (13.9 vs. 29.1%) (see Figure 3 and Table 1).

**TABLE 1:** Demonstratives by distance and hearer position for Spanish speakers living in Spain.

			**Demonstrative**			**Total**
			**Proximal term**	**Medial term**	**Distal term**	
Side-by-side	Distance	25 cm	87	3	0	90
		50 cm	83	7	0	90
		150 cm	2	83	5	90
		175 cm	1	78	11	90
		275 cm	0	22	68	90
		300 cm	0	20	70	90
	Total		173	213	154	540
Opposite	Distance	25 cm	85	5	0	90
		50 cm	84	6	0	90
		150 cm	3	80	7	90
		175 cm	2	78	10	90
		275 cm	0	19	71	90
		300 cm	0	18	72	90
	Total		174	206	160	540

**FIGURE 4:**
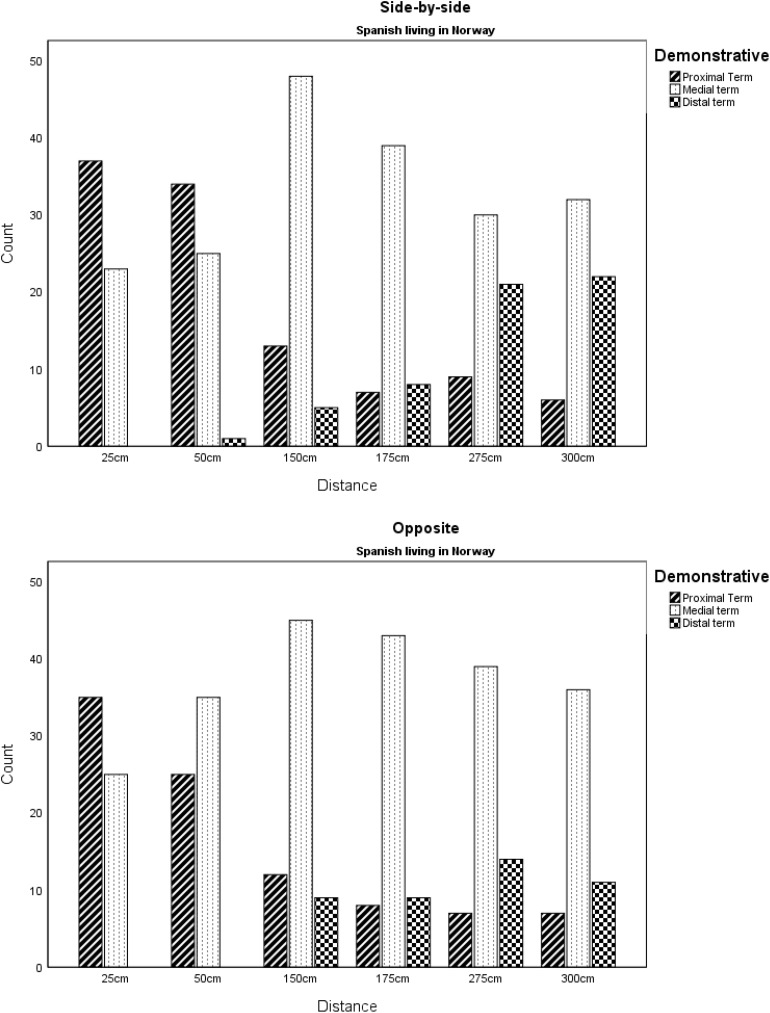
Demonstratives by distance and hearer position for Spanish speakers living in Norway.

In the *Spanish Living in Norway* (SLiN) group there were also 29 occasions when participants used *este* in the 275 and 300 cm positions. These were seen both when the listener was side-by-side or opposite, against zero occurrences of *este* in the *Spanish Living in Spain* (SLiS) group in the 275 and 300 cm positions. Examination of the data showed that 23 of the 29 uses of *este* at 275 or 300 cm were attributable to two individuals (11 times and 12 times apiece), four other individuals used it once, and one further individual used it twice. In line with the hypothesis about time spent living in Norway as a predictor for different use of Spanish demonstratives, the use of *este* at 275 or 300 cm was tabulated alongside time living in Norway. Initial inspection of the data suggests longer exposure to the L2 measured in terms of length living in Norway was not associated with this different use of *este* by these two individuals (note: the median time living in Norway for the whole sample is 84 months, min 3 months, max 444 months) (see Figure 4 and Table 2).

**TABLE 2:** Demonstratives by distance and hearer position for Spanish speakers living in Norway.

			**Demonstrative**			**Total**
			**Proximal term**	**Medial term**	**Distal term**	
Side-by-side	Distance	25 cm	37	23	0	60
		50 cm	34	25	1	60
		150 cm	13	48	5	66
		175 cm	7	39	8	54
		275 cm	9	30	21	60
		300 cm	6	32	22	60
	Total		106	197	57	360
Opposite	Distance	25 cm	35	25	0	60
		50c m	25	35	0	60
		150 cm	12	45	9	66
		175 cm	8	43	9	60
		275 cm	7	39	14	60
		300 cm	7	36	11	54
	Total		94	223	43	360

### Regression Models

For the analysis we carried out multilevel regression models which allow for the inter-related variance within all responses within a level, such as correlations within the responses of one individual, and possibly within the responses of individuals of one language compared to another. These variances are reported in the Random effect part of the model. The independent predictor variables are reported through the Fixed Effects. The models are all multinomial with LOGIT link, with the following three reference categories: the proximal term, side-by-side hearer position, and 25 cm distance.

We ran 2 models. Model 1a and 1b had language as level 1 (variety of language, i.e., Spanish Living in Norway and Spanish) and ID (individuals) as level 2. The two fixed effect predictors were position of hearer and distance. The two-way interaction of position of hearer x distance was not significant in Model 1a [*F*(10, 1776) = 1.082, *p* = 0.372], and was thus removed for Model 1b, which was the final model for the two level with interaction MLM analysis. Model 1b [*F*(12, 1786) = 49.379, *p* ≤ 0.001] correctly predicted 89.6% of demonstratives, with significant fixed effects for distance [*F*(2, 1786) = 59.201, *p* ≤0.001) and position of hearer [*F*(2, 1786) = 3.426, *p* = 0.033]. However, running model 1 showed that the amount of variance explained by language (level 1) was non-significant (Z = 0.562, *p* = 0.574 for medial and Z = 0.579, *p* = 0.563 for distal), though the variance accounted for by individuals within each language (level 2) was significant (*Z* = 3.836, *p* ≤ 0.001 for medial and Z = 3.994, *p* ≤ 0.001 for distal). For this reason, we amended the model to a one level model with just the variance within individuals’ responses accounted for as a “level” in Model 2 (see Table 3).

**TABLE 3:** Model 1b—Demonstrative by distance and hearer position with two levels (language and individual).

**Fixed coefficients**								
		**Coefficient**	**Std. error**	***t***	**Sig**	**Exp (Coefficient)**	**95% Confidence interval for Exp (Coefficient)**	
							**Lower**	**Upper**
Medial term	Intercept	–6.642	1.1986	–5.541	< 0.001	0.001	< 0.001	0.014
	Opposite	0.063	0.2636	0.241	0.810	1.066	0.635	1.787
	Distance 300 cm	9.431	0.9063	10.405	< 0.001	12462.911	2106.910	73721.315
	Distance 275 cm	9.185	0.8745	10.503	< 0.001	9753.152	1754.807	54207.652
	Distance 175 cm	9.790	0.8272	11.836	< 0.001	17863.048	3526.983	90470.660
	Distance 150 cm	9.077	0.7851	11.563	< 0.001	8754.758	1877.356	40826.462
	Distance 50 cm	1.458	0.7261	2.008	0.045	4.297	1.034	17.851
Distal term	Intercept	–3.383	1.1417	–2.963	0.003	0.034	0.004	0.319
	Opposite	0.462	0.2475	1.867	0.062	1.587	0.977	2.580
	Distance 300 cm	8.488	0.6499	13.060	< 0.001	4855.323	1357.167	17370.124
	Distance 275 cm	8.060	0.6050	13.324	< 0.001	3166.541	966.709	10372.288
	Distance 175 cm	6.015	0.5407	11.123	< 0.001	409.463	141.781	1182.525
	Distance 150 cm	5.107	0.4721	10.818	< 0.001	165.188	65.441	416.969
	Distance 50 cm	0.746	0.3490	2.139	0.033	2.110	1.064	4.183

*Reference: Proximal term, side-by-side hearer position, 25 cm distance.*

Model 2a, 2b, 2c and 2d had language as a predictor and ID (individuals) as the only level. The three fixed effect predictors were language, position of hearer and distance. All interactions are first entered and then higher order interactions removed if not significant. The three-way interaction of language x position of hearer x distance was non-significant in Model 2a [*F*(10, 1752) = 0.396, *p* = 0.949], and was removed for Model 2b, then the non-significant two-way interaction position of hearer x distance [*F*(10, 1762) = 0.781, *p* = 0.648] was removed for Model 2c, and then the non-significant two- way interaction position of hearer x language [*F*(2, 1772) = 0.573), *p* = 0.573] was removed for Model 2d, which is the final model for the single level with interaction MLM analysis. Model 2d [*F*(24, 1774) = 24.745, *p* < 0.001 correctly predicted 86.7% of demonstratives correctly with significant fixed effects for distance [*F*(10, 1774) = 16.881, *p* < 0.001] and for the language x distance interaction [*F*(10, 1774) = 21.456], and not significant for language [*F*(2, 1774) = *p* = 0.994] and for position of hearer [*F*(2, 1774) = 2.798, *p* = 0.061]. The variance accounted for by level 1 (individuals within each language) was significant (*Z* = 3.044, *p* = 0.002 for medial and *Z* = 3.075, *p* = 0.002, for distal) (see Table 4).

**TABLE 4:** Model 2d—Demonstrative by language, distance and hearer position with one level (individual).

**Fixed coefficients^*a*^**								
**Demonstrative**		**Coefficient**	**Std. error**	***t***	**Sig.**	**Exp (Coefficient)**	**95% Confidence interval for Exp (Coefficient)**	
							**Lower**	**Upper**
Distal term	Intercept	–1.306	0.4852	–2.691	0.007	0.271	0.105	0.702
	Opposite	0.355	0.2124	1.670	0.095	1.426	0.940	2.163
	Distance 300 cm	3.271	0.4962	6.592	< 0.001	26.343	9.953	69.719
	Distance 275 cm	2.937	0.4589	6.400	< 0.001	18.862	7.668	46.399
	Distance 175 cm	3.269	0.4635	7.053	< 0.001	26.297	10.594	65.272
	Distance 150 cm	2.695	0.3920	6.874	< 0.001	14.799	6.860	31.923
	Distance 50 cm	0.617	0.3177	1.943	0.052	1.854	0.994	3.457
	Language	–16.820	324.8142	–0.052	0.959	49.6E-9	1.057E-284	232.5E + 267
	Language*300 cm	32.791	449.8260	0.073	0.942	174.1E + 12	< 0.001	
	Language*275 cm	33.090	449.7033	0.074	0.941	234.9E + 12	< 0.001	
	Language*175 cm	17.587	324.8152	0.054	0.957	43.4E + 6	9.242E-270	204.1E + 282
	Language*150 cm	16.974	324.8150	0.052	0.958	23.5E + 6	5.011E-270	110.5E + 282
	Language*50 cm	–0.575	459.4176	–0.001	0.999	0.563	< 0.001	
Third term	Intercept	–17.483	402.8104	–0.043	0.965	2.554E-08	< 0.001	
	Opposite	–0.007	0.2336	–0.030	0.976	0.993	0.628	1.570
	Distance 300 cm	18.624	402.8104	0.046	0.963	122.5E + 6	< 0.001	
	Distance 275 cm	18.351	402.8104	0.046	0.964	93.3E + 6	< 0.001	
	Distance 175 cm	17.447	402.8104	0.043	0.965	37.8E + 6	< 0.001	
	Distance 150 cm	16.399	402.8104	0.041	0.968	13.2E + 6	< 0.001	
	Distance 50 cm	12.284	402.8114	0.030	0.976	216.2E + 3	< 0.001	
	Language	13.421	402.8103	0.033	0.973	674.3E + 3	< 0.001	
	Language*300 cm	1.741	509.0141	0.003	0.997	5.704	< 0.001	
	Language*275 cm	2.128	508.9057	0.004	0.997	8.402	< 0.001	
	Language*175 cm	–7.771	402.8112	–0.019	0.985	< 0.001	< 0.001	
	Language*150 cm	–7.242	402.8110	–0.018	0.986	0.001	< 0.001	
	Language*50 cm	–11.716	402.8117	–0.029	0.977	8.166E-06	< 0.001	

*Reference: Proximal term, side-by-side hearer position, 25 cm distance, Spanish in Spain. Link function: Generalized logit^*a*^.*

In a separate model we analyzed only the data from the Spanish Living in Norway group, in order to assess the effect of time spent (i.e., exposure to the L2) in Norway on their performance. Time spent in Norway was entered as a random effect, and turned out to be highly non-significant (*p* = 0.926).

The Norwegian native speaker group was not included in the multilevel regression models due to lack of comparable number of dependent variables (two vs. three deictic terms). The descriptive data from that group, nevertheless, revealed an overwhelming use of the distal term [*den der* (that (over) there)] for all positions (689 times, 83.2%), except for the closest distances (25 and 50 cm) (139 occurrences, 16.8%), which were named by the proximal term *den her* (this here).

## Discussion and Final Remarks

In the current study, we expected the group of Spanish native speakers living in Norway to perform comparably to the control group of native speakers living in Spain. This was driven by theoretical accounts and hypotheses of language attrition, which is assumed to affect less robust systems first, leaving early acquired, basic and more robust systems relatively intact (Jakobson, 1941; Keijzer, 2010). This main hypothesis was not borne out. We saw a dramatic difference in the use of the three terms available in Spanish between the two groups. While the SLiS group used the three terms according to classical descriptions of the language, and previous experimental research (Coventry et al., 2008), the SLiN group saw a dramatic drop of the distal term (*aquel*), combined with an overwhelming use of the medial (third) term *ese*. The latter was used across the board for all experimental distances, and even in place of *este* for the closest object locations, with an equal number of *este* and *ese* already for the 50 cm distance. The regression analysis in Model 2d further confirmed the difference between the two groups of speakers through the significant language x distance interaction.

These results suggest that *ese* is becoming a neutral deictic term appropriate for referring to all possible locations of the referent with regard to the deictic center. This is true for Spanish native speakers who have moved to another country (Norway), which features a deictic system different from the Spanish one. Interestingly, this convergence on a two-term system, whereby the proximal term (*este*) is reserved for locations in the immediate vicinity of the speaker, and a second, neutral term (*ese*), is used to refer deictically to other and further locations beyond this one, is highly reminiscent of the results from the native Norwegian group (see also Coventry, in preparation). Two possible accounts present themselves. One possibility is that the observed change in deictic term use is the result of cross-linguistic transfer, leading to, sometimes irreversible, changes in the L1 language system, i.e., attrition (Cook, 2003; Köpke and Schmid, 2004). However, bi-directional influence of the two languages of the bilingual has been recognized in all traditions studying language learning and processing. Thus, the current results can also be attributed to the effects of bilingual language usage (Grosjean, 1992; Kroll and Bialystok, 2013). Following Schmid and Köpke (2007), we believe that the two perspectives are reconcilable and not mutually exclusive. It is thus possible that the observed results are attributable to a bilingual system of mapping perceptual space onto the native language (Spanish), primarily reflected in language use, and as a result of daily practice of a second language. Indeed, recent studies on attrition in Spanish speakers exposed to English document that attrition effects may be partly reversible when speakers are re-immersed in the original L1 community (Chamorro et al., 2016; Chamorro and Sorace, 2019). These findings indicate that bilingual grammars are dynamic systems which reflect sensitivity to frequency of use. It may be further speculated that it is not the grammar itself that shows irreversible changes in first-generation speakers, but rather access to the grammar and the flexibility to map linguistic labels to referents in context. Since mapping between demonstrative form and contextual features which impact on deictic use requires cognitive effort, bilinguals may not always be in a position to do the appropriate mapping (Sorace, 2011, 2016, 2020). This may result in simplification and overuse of the most neutral or explicit form which fits a wider range of referential contexts, indicative of adaptive changes as a result of bilingual exposure (Sorace, 2016).

Simplification has been documented in other domains of first-generation language use. For example, the study by Tsimpli et al. (2004) provides evidence of attrition of subject pronouns in native speakers of Italian, a null-subject language after prolonged exposure to English. This study shows a selective simplification of the original system with inappropriate extension of the explicit form, in parallel with evidence from L2 speakers of such languages. Research on adult and child bilingual speakers of two null-subject languages of the same type found the same over-extension of the overt pronoun (Malgaza and Bel, 2006; Bonfieni et al., 2019).

Our results further suggest that deictic referential systems may “shrink” over time, and under pressure from bilingual language exposure, when certain perceptual distinctions are no longer systematically encoded in the respective terms. This is evidenced by diachronic changes in many Indo-European languages, whereby three-term systems evolve into two-term systems (Frei, 1944; Lyons, 1999; Manolessou, 2002; Vulchanova and Vulchanov, 2011). Interestingly, the Spanish living in Norway group appear to have converged on the medial (third) term (*ese*) as a distance-neutral term appropriate for reference to all types of locations relative to speaker, even including the peri-personal space, where the proximal term *este* is in close competition with this neutral term. Thus, at the 25 cm location, *este* was used a total of 72 times, against 48 for *ese*, while at 50 cm the two terms are already used equally often (59 vs. 60). Yilmaz and Schmid (2018) attribute the subsequent changes in L1 attrition exactly to an initial process of competition between items. Furthermore, a similar development has been attested also diachronically in the history of Bulgarian where the neutral term has come to replace the proximal one over time, subsequently becoming grammaticalized as an article (Vulchanova and Vulchanov, 2010, 2011). From a psycholinguistic and diachronic perspective, however, an open question remains whether to treat phenomena of this type as just a simplification or rather as a re-organization in the mapping of form to meaning, whether irreversible or dynamic.

Surprisingly, in a separate analysis run only on the SLiN group, time spent in Norway was highly non-significant. This finding is unexpected given the role of length of stay in host country, which is typically used as an important inclusionary criterion in attrition research. However, it is consistent with an account of deictic term use as driven by universal cognitive principles and parameters, rather than language-specific constraints and lexical encoding (Coventry et al., 2014), as well as with the changes documented in language diachrony discussed above. Furthermore, given these results, and the prevalence of two-term systems in the survey in Diessel (2005, 2013), it may be stipulated that three-term systems are less stable than two-term systems, by lexicalizing more, and more subtle distinctions.

An interesting finding in the current study is the absence of impact of position of hearer. Results for term use did not differ significantly between the two experimental conditions, and between the two groups of participants, also confirmed by the lack of significant effect of position of hearer. This result is unexpected against the semi-naturalistic performance results and account provided in Jungbluth (2003) and in Jungbluth and Da Milano (2015), where face-to-face deictic reference was dictated by a person-oriented system. Also, on that account, speakers are expected to differ as a result of the face-to-face constellation on use of the distal term *aquel*, but not on the proximal one (*este*). However, the native Speakers Living in Spain (SLiS) in our study used an equal number of distal terms between the two conditions for the relevant distance locations (275 and 300 cm). The current results contradict also the findings in Coventry et al. (2008), where position of hearer impacted on the use of the deictic terms available in Spanish, and interacted with distance. In the current study, the interaction between position of hearer and distance was non-significant, as was the interaction with language, for both groups of speakers. Given that no other differences with this earlier study of Spanish were evident in our results, and the descriptive data in both studies are highly consistent, we attribute the current finding to a methodological difference. Coventry et al. (2008) found a main effect of position of hearer only for the proximal term *este*, and an interaction with distance again only for *este*, whereby use of *este* was affected exclusively in the intermediate object positions at 100, 125, and 150cm. In the current design these positions were not named by participants, except for the 150 cm distance, and thus no data were correspondingly included in the analyses, explaining why this subtle interaction was not documented. If anything, we see a reduced use of proximal *este* in the 50 cm object location (56 vs. 41%), against an increase of *ese* (41 vs. 58%) when the hearer is seated opposite the participant, and only in the Spanish Living in Norway group, consistent with their overall preference for *ese*.

Overall, the current results indicate that peri-personal space is an important parameter in the mapping of perceptual space onto language, and are, as such, consistent with extant research and ideas on deictic demonstrative use (Coventry et al., 2014; Caldano and Coventry, 2019; Peeters et al., 2020). Thus, across both groups of Spanish participants in the study, as well as the Norwegian native group, locations closest to the speaker (25 and 50 cm), and within arm length’s reach, were primarily associated with use of the respective proximal terms. The differences between groups arose first with respect to reference to locations outside of this region. The finding that the Spanish native speakers living in Norway are converging on a relatively simpler system, based on a proximal term (*este*), and a neutral term (*ese*) which is used for all other locations, further confirms this idea. These results are consistent with, and further support, Diessel (2014) suggestion that spatial specifications are still relevant for the semantic analysis of demonstratives.

The current study fills a gap in research on deictic use under conditions of immersive exposure to a second language, and specifically, on possible changes the L1 deictic reference system can undergo under bilingual pressure.

## Data Availability Statement

The raw data supporting the conclusions of this article will be made available by the authors, without undue reservation.

## Ethics Statement

The studies involving human participants were reviewed and approved by the Norwegian Data Protection Service (NSD), Comité de Ética de la Investigación (Universidad de Islas Baleares), and the School of Psychology Ethics Committee at the University of East Anglia. The participants provided their written informed consent to participate in this study.

## Author Contributions

MV and VV managed the data collection in Norway and prepared the manuscript. PG-F managed the data collection in Spain and provided the description of the Spanish demonstrative system. JC ran the statistical analyses. MV, VV, JC, and PG-F edited the manuscript for submission. All authors contributed to the article and approved the submitted version.

## Conflict of Interest

The authors declare that the research was conducted in the absence of any commercial or financial relationships that could be construed as a potential conflict of interest.
